# Drug-resistant clinical *Candida* responded to 5-aminolevulinic acid photodynamic therapy (ALA-PDT) *in vitro* via enhanced ROS release

**DOI:** 10.1128/aac.01645-25

**Published:** 2026-04-17

**Authors:** Zhiya Yang, Yonghong Zhang, Dongmei Li, Sisi Wang, Ruoyu Shi, Dongmei Shi

**Affiliations:** 1 The Laboratory of Medical Mycology, Jining No. 1 People's Hospital 117947 , Jining, Shandong, China; 2 Department of Dermatovenereology, West China Hospital, Sichuan University 12530, Chengdu, China; 3 Shandong University of Traditional Chinese Medicine 74738, Jinan, Shandong, China; 4 Department of Microbiology and Immunology, Georgetown University Medical Center 12231, Washington, DC, USA; 5 Department of Dermatology, Jining No. 1 People’s Hospital 117947 , Jining, Shandong, China; University Children's Hospital Münster, Münster, Germany

**Keywords:** 5-aminolevulinic acid-photodynamic therapy (ALA-PDT), *Candida*, reactive oxygen species (ROS), scanning electron microscope (SEM), mycelia-related genes

## Abstract

Photodynamic therapy (PDT) has emerged as a potential strategy to combat drug-resistant fungal infections. This study aimed to evaluate the therapeutic efficacy of 5-aminolevulinic acid PDT (ALA-PDT) against drug-resistant *Candida* strains isolated from patients with candidiasis and to elucidate its underlying mechanisms. Drug-resistant strains were identified by their reduced antifungal susceptibility to azoles, micafungin, amphotericin B, and terbinafine. RNA sequencing (RNA-seq) was used to assess gene expression related to mycelial formation and fungal growth. Scanning electron microscopy (SEM) was employed to observe cell-wall morphology after ALA-PDT treatment. Biofilm formation and intracellular reactive oxygen species (ROS) levels were also measured to investigate the mechanism of ALA-PDT-mediated fungal killing. *Candida albicans* and *Candida tropicalis* accounted for over half, predominantly from male ICU patients aged over 40 years. *Candida* spp. strains exhibited resistance rates of approximately 69.38% to terbinafine, 40.8% to azole antifungals, and 16.3% to micafungin among 49 drug-resistant isolates. Treatment with 20% ALA-PDT effectively eradicated those azole-resistant *C. albicans in vitro*, increased fungal killing, and significantly increased intracellular ROS levels. SEM analysis revealed varying degrees of cell-wall disruption, and RNA-seq demonstrated downregulation of mycelia-related genes following ALA-PDT. ALA-PDT effectively inhibits drug-resistant *Candida* spp. growth by inducing ROS-mediated cell damage and suppressing mycelial gene expression. These findings highlight ALA-PDT as a promising therapeutic approach for refractory candidiasis and provide mechanistic insights into its antifungal activity.

## INTRODUCTION

Candidiasis, caused by *Candida* species, poses significant clinical challenges across diverse medical fields, ranging from superficial mucosal infections to severe, life-threatening systemic diseases, particularly in immunocompromised patients (1, 2). Antifungal agents remain the primary therapeutic option for candidiasis (3). However, the extensive and prolonged use of these agents has led to a marked rise in drug-resistant *Candida* species strains in clinical settings. Moreover, the intrinsic ability of *Candida* species to form biofilms further complicates treatment, as biofilm-associated cells exhibit markedly higher resistance to antifungal agents compared to planktonic cells (4).

To date, more than 15 *Candida*-related species have been implicated in human infections, among which *Candida albicans* (*C. albicans*) remains the predominant pathogen (5, 6). It is estimated that *C. albicans* accounts for nearly 60% of invasive fungal infections (7).

Antifungal drugs remain the main treatment for candidiasis, but their long-term and extensive use has led to a significant increase in drug-resistant *Candida* strains in clinical practice. The core drug resistance mechanisms mainly include overexpression of efflux pumps such as CDR1 and MDR1 gene-mediated drug efflux, target mutations such as ERG11 gene mutations corresponding to azole drugs and FKS1/FKS2 gene mutations corresponding to echinocidins, and overexpression of target proteins. It is worth noting that the inherent biofilm-forming ability of *Candida* plays a key cofactor role in this drug resistance cascade reaction. The strong biofilm-forming capacity of *C. albicans* contributes to its enhanced resistance to commonly used antifungal agents (8, 9), leading to frequent recurrences and increased mortality rates. Therefore, there is an urgent need to develop innovative therapeutic strategies capable of effectively controlling drug-resistant *Candida* spp. and overcoming the limitations of conventional antifungal therapy.

5-Aminolevulinic acid photodynamic therapy (ALA-PDT) is an emerging, non-invasive therapeutic modality that combines a photosensitizer (5-aminolevulinic acid, ALA) with light irradiation to generate reactive oxygen species (ROS) through photodynamic reactions, resulting in selective cytotoxicity toward target cells (10). Recent studies have demonstrated that ALA-PDT can effectively inhibit drug-resistant *C. albicans* and suppress its biofilm formation (11, 12). However, the precise mechanisms underlying ALA-PDT–mediated killing of drug-resistant *Candida* spp. strains remain largely unclear. Due to the limitation of penetration depth, ALA-PDT therapy is mainly used to treat superficial lesions, such as skin diseases and mucosal lesions. In this study, our aim is to evaluate the *in vitro* experiments of ALA-PDT on the inhibition of *Candida* spp. biofilms and explore the related mechanisms.

In the present study, we performed a comprehensive analysis of clinical data and *Candida* species isolates from patients diagnosed with candidiasis across multiple departments in a tertiary-grade (Grade A) hospital. Drug-resistant *Candida* spp. strains were isolated and subjected to ALA-PDT to assess its antifungal efficacy and underlying mechanisms. Our findings reveal that ALA-PDT effectively eradicates drug-resistant *C. albicans in vitro*, primarily through the induction of intracellular ROS accumulation and subsequent cell wall disruption. These results suggest that ALA-PDT represents a promising adjunct or alternative approach for managing drug-resistant *C. albicans* infections and biofilm-associated candidiasis.

## MATERIALS AND METHODS

### ALA-PDT treatment of drug-resistant *C. albicans in vitro*

In our previous study, 206 *Candida* spp. strains were isolated from candidiasis patients and tested for antifungal susceptibility, leading to the acquisition of 49 drug-resistant strains (13).

Drug-resistant *C. albicans* strains were subcultured on Sabouraud dextrose agar (SDA) at 37 °C for 24 h. *Conidia* were harvested into phosphate-buffered saline (PBS) and allowed to settle for 3–5 min. The supernatant was carefully transferred to a sterile tube and vortexed thoroughly.

Each isolate was divided into 15 experimental groups: an untreated control group, seven groups treated with various concentrations of ALA-PDT (20%, 10%, 5%, 2.5%, 1.25%, 0.625%, and 0.313% ALA) under a constant light intensity of 100 mW/cm², and seven parallel groups exposed to ALA without light irradiation.

ALA stock solution (40% m/vol) was prepared by dissolving ALA powder (Fudan Zhangjiang, Shanghai, China) in sterile water and diluted to the desired working concentrations. In a 96-well microplate, 5 × 10⁴ colony-forming unit (CFU) fungal cells in 100 µL of medium were mixed with 100 µL of ALA solution and incubated in the dark for 3 h. For the PDT treatment groups, samples were irradiated with a 635 nm LED light source (Omnibus, UK) at 100 mW/cm² for 20 min, with the light source positioned 4 cm above the plate surface. After irradiation, cultures were incubated at 37°C for 24–48 h. Control groups included fungi only, ALA only, and medium only. Detailed experimental procedures have been described in our previous publication (14).

### Effects of ALA-PDT on morphological and biofilm changes

The effects of ALA-PDT on the morphology and biofilm formation of drug-resistant *C. albicans* were compared with those of drug-sensitive strains. Yeast cells in the logarithmic growth phase were re-suspended in 5 mL of RPMI-1640 medium and adjusted to a concentration of 1 × 10⁵ CFU/mL. The suspensions were treated with 20% ALA-PDT and incubated on a constant-temperature shaker at 30°C and 200 rpm for 4 h.

The growth and morphological transition between blastoconidium and hyphae were monitored at 0, 4, 8, and 12 h post–ALA-PDT treatment. At the 12 h endpoint, cells were collected by centrifugation at 12,000 × *g* for 1 min and stained with calcofluor white (CFW) in PBS. Fungal morphology, including blastoconidia and mycelial development, was observed under a fluorescence microscope. In parallel, cells were fixed with pre-chilled 2.5% (wt/vol) glutaraldehyde at 4°C for scanning electron microscopy (SEM). Samples underwent standard graded acetone dehydration, critical-point drying, and gold sputter-coating prior to imaging. SEM micrographs were acquired to evaluate ultrastructural alterations of the cell membrane and biofilm organization following ALA-PDT, as described previously (15).

### Fungal killing after ALA-PDT treatment

Azole-resistant *C. albicans* strains were selected for combined antifungal effect with ALA-PDT, as azole resistance is most frequently encountered in this species (16, 17). Following ALA-PDT treatment, 100 μL of the final conidial suspension was transferred into each well of 96-well microtiter plates for antifungal susceptibility testing. The plates were incubated at 37°C for 24–48 h, after which fungal growth was visually assessed.

To evaluate the fungicidal efficacy of ALA-PDT, 2 μL aliquots of each conidial suspension were inoculated onto Sabouraud Dextrose Agar (SDA) plates and incubated at 37°C for 24 h. Colony-forming units (CFUs) were counted to determine fungal survival rates following ALA-PDT exposure.

### Colocalization of mitochondria and ALA-PDT–induced porphyrins in *C. albicans*

The intracellular localization of protoporphyrin IX (PpIX) following ALA-PDT treatment was examined using laser scanning confocal microscopy (LSCM). PpIX generated by intracellular metabolism of ALA exhibits red fluorescence, enabling visualization of its accumulation within fungal cells.

Mitochondria were labeled using MitoTracker Green FM dye (MCE, CAS No. 201860-17-5). Briefly, *C. albicans* cells were incubated with 500 nM MitoTracker Green FM diluted in pre-warmed, serum-free RPMI-1640 medium (400 µL per sample) for 35 min at 37°C. After staining, cells were centrifuged at 1,000 × *g* for 3 min, and the supernatant was discarded. The nuclei were subsequently stained with DAPI for 5 min, followed by three washes with phosphate-buffered saline (PBS).

Colocalization of mitochondria (green fluorescence) and ALA-induced PpIX (red fluorescence) was visualized using LSCM (Leica Microsystems, Germany). The degree of colocalization was analyzed qualitatively to assess mitochondrial involvement in ALA metabolism and photodynamic response.

### Total reactive oxygen species (ROS) measurement in *C. albicans* following ALA-PDT treatment

Total intracellular ROS levels were quantified using a 2′,7′-dichlorofluorescein diacetate (DCFH-DA) assay kit (Beyotime Biotechnology, S0033S) following the manufacturer’s protocol. After ALA-PDT treatment, *C. albicans* conidial suspensions were incubated with 2′,7′-dichlorofluorescein diacetate (DCFH-DA) at a final concentration of 10 µM for 20 min at 37°C in the dark (18).

Following incubation, cells were washed gently with phosphate-buffered saline (PBS) to remove excess dye. Fluorescence intensity was then measured using a microplate reader at an excitation wavelength of 490 nm and an emission wavelength of 520 nm. All experiments were performed in triplicate, and ROS production was expressed as relative fluorescence intensity compared to untreated controls.

### Assessment of membrane permeability in *C. albicans* under antifungal drugs and/or ALA-PDT

Changes in membrane permeability of *C. albicans* were evaluated using the propidium iodide (PI) uptake assay. PI is impermeable to intact cell membranes but can penetrate cells with compromised membranes, binding to nuclear DNA and emitting fluorescence.

Four experimental groups were established: (i) untreated control, (ii) antifungal drug treatment, (iii) ALA-PDT treatment, and (iv) combined antifungal drug and ALA-PDT treatment. Logarithmic-phase *C. albicans* cells were adjusted to 1 × 10⁵ CFU/mL, treated accordingly, and incubated on a constant-temperature shaker at 30°C and 180 rpm for 12 h. Cells were subsequently washed with sterile phosphate-buffered saline (PBS) and incubated with 10 µg/mL PI in PBS at room temperature for 30 min in the dark. Excess dye was removed by washing with PBS, and fluorescence images were captured using a fluorescence microscope to assess membrane integrity (19).

### RNA extraction and Western blot analysis

Total RNA was extracted from ALA-PDT–treated and control *C. albicans* samples for transcriptome analysis. RNA sequencing (RNA-seq) was performed on an Illumina NovaSeq 6000 platform to identify differentially expressed genes. Genes related to mycelial formation were further validated by real-time quantitative PCR (RT-qPCR) (20). RT-qPCR was conducted using specific primers (listed in Table 1) on a Bio-Rad CFX96 system with SYBR Green dye, and each sample was analyzed in triplicate. Melting curve analysis was performed to verify the specificity of PCR products. Relative mRNA expression levels were calculated using the 2^−ΔΔCt^ method (21).

**TABLE 1:** Primers used for RT-PCR

Gene	PCR primer
ACT1	Forward: TTTGCCGGTGACGACGCTCC
	Reverse: CGTCCCAGTTGGAAACAATACCGTGT
HGC1	Forward: TACACCAGGTCGCAAGCAACAAC
	Reverse: AACAGCACGAGAACCAGCGATAC
HWP1	Forward: ACCCACAACAACAACCACAAGAGC
	Reverse: TTGAGGTGGATTGTCGCAAGGTTC
NRG1	Forward: ACGGTGGTTGCACGTTGTCG
	Reverse: TGCTGCTGCTGCTTGGTTGGT
EFG1	Forward: CATCACAACCAGGTTCTACAACCAAT
	Reverse: CTACTATTAGCAGCACCACCC
YBP1	Forward: GCCGCATACTTGAATCTATT
	Reverse: AAGCATACCAGCAGCATT
POX18	Forward: GATTGCACTCGGCACAGTTAAAGC
	Reverse: TTTGGTGGTGTTTGCTCGGACTC
SNO1	Forward: ACCCGGACGAATATGCAGTGTATG
	Reverse: CTGCTTTCTCCGCCAGGAATAACC
RFG1	Forward: AACAGCAACAGCAGCAACAACAAG
	Reverse: ACCTCCACCTCCACCTCCATTAAG
TUP1	Forward: TCAGTCTACTCTGTCGCCTTCTCC
	Reverse: AGTGCCACAACTTGACGGTTCTG

For protein analysis, cells were lysed in RIPA buffer containing 0.2 mM PMSF, 1 mM EDTA, and 1 μg/mL phosphatase inhibitors for 10 min on ice. Lysates were centrifuged, and the supernatants were collected for Western blotting.

Proteins were separated by SDS-PAGE, transferred to PVDF membranes, and probed with antibodies against P38 and cleaved-caspase-3. Glyceraldehyde-3-phosphate dehydrogenase was used as the internal loading control.

### Statistical analysis

Data are presented as mean ± standard deviation from at least three independent experiments. Statistical comparisons among groups were performed using one-way analysis of variance in GraphPad Prism 8.0. A *P*-value < 0.05 was considered statistically significant.

## RESULTS

### ALA-PDT significantly inhibited drug-resistant *C. albicans* growth in *vitro*

**Fig 1:**
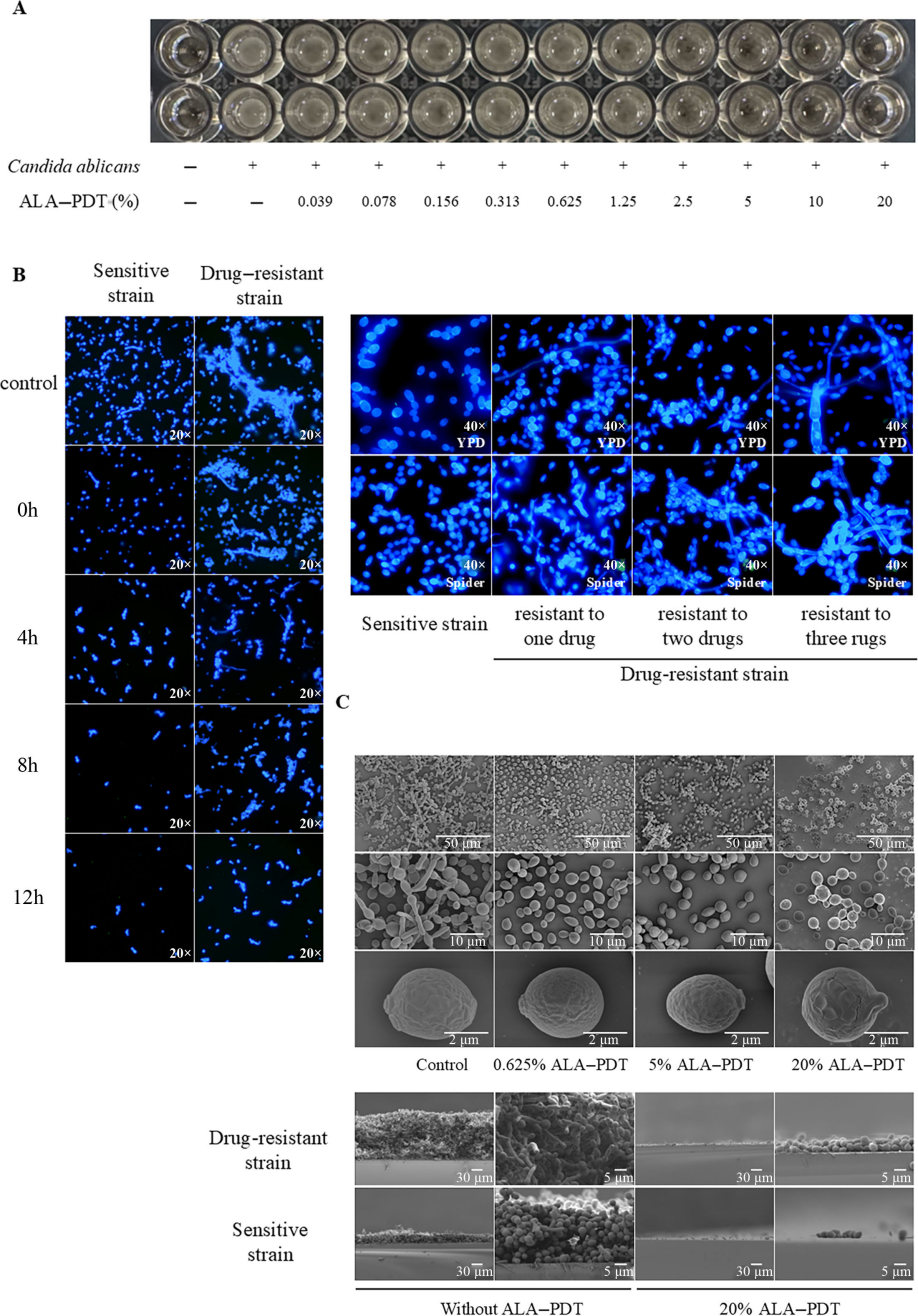
5-Aminolevulinic acid photodynamic therapy (ALA-PDT) inhibits blastoconidia and mycelial growth of *Candida albicans*. (**A**) 20% ALA-PDT treatment inhibited blastoconidia growth in drug-sensitive *C. albicans* and partially inhibited mycelial growth in drug-resistant *C. albicans* over 12 h. (**B**) Comparison of sensitive and drug-resistant *C. albicans* in mycelial induction media (Spider and yeast extract peptone dextrose [YPD]) at 12 h post-ALA-PDT treatment. Sensitive strains showed reduced blastoconidia germination, while resistant strains exhibited shorter mycelia. (**C**) Scanning electron microscopy images showing cell morphology and mycelial inhibition in drug-resistant *C. albicans* treated with 0, 0.625%, 5%, or 20% ALA-PDT. Higher ALA-PDT concentrations induced obvious cell membrane changes and inhibited mycelial formation. No significant difference in biofilm thickness was observed between sensitive and resistant strains following ALA-PDT treatment.

To evaluate the effect of ALA-PDT on drug-resistant *C. albicans*, various concentrations of ALA were tested to determine the optimal working concentration for inhibiting fungal growth. ALA-PDT treatment significantly suppressed *C. albicans* proliferation compared to untreated controls. Notably, 20% ALA-PDT exhibited the strongest fungicidal effect, whereas concentrations below 20% only partially inhibited growth (Fig. 1A).

The effect of 20% ALA-PDT on mycelial germination and blastoconidia proliferation was assessed using calcofluor white (CFW) staining at 0, 4, 8, and 12 h in both drug-susceptible and drug-resistant strains. Drug-resistant strains initially displayed higher blastoconidia counts than susceptible strains. Following 20% ALA-PDT treatment, the growth rate of drug-resistant strains decreased more prominently than that of sensitive strains. However, quantitative analysis based on three independent experiments demonstrated that ALA-PDT significantly reduced blastoconidial counts in both drug-susceptible and drug-resistant strains compared with their respective untreated controls (Fig. 1D).

Further analysis compared mycelial growth among single-, double-, and triple-drug resistant strains and sensitive strains after 20% ALA-PDT treatment. Three-drug resistant strains exhibited the highest mycelial growth, followed by double- and single-drug resistant strains, while sensitive strains showed no mycelial germination throughout the treatment and maintained blastoconidia morphology (Fig. 1B). These results suggest that drug-resistant strains germinate more rapidly than drug-sensitive strains under untreated conditions, but this rapid growth can be effectively suppressed by high-concentration ALA-PDT treatment, highlighting its potential against resistant *C. albicans*.

### Scanning electron microscopy revealed altered cell membrane morphology and biofilm formation following ALA-PDT treatment

Scanning electron microscopy (SEM) was used to examine changes in the cell membrane of *C. albicans* treated with different concentrations of ALA-PDT. As shown in Fig. 1C, blastoconidia in the untreated control group were elliptical or round, accompanied by mycelial formation, with clear cell membrane outlines and regular morphology. Treatment with 0.625%, 5%, or 20% ALA-PDT did not significantly alter blastoconidia morphology compared to the control, but mycelial production was progressively reduced with increasing ALA-PDT concentration. Notably, the 20% ALA-PDT treatment group exhibited obvious membrane surface cracks, indicating a direct effect of high-concentration ALA-PDT on membrane integrity.

When comparing biofilm formation between sensitive and drug-resistant strains after ALA-PDT treatment, we observed that drug-resistant strains predominantly exhibited mycelial growth, whereas sensitive strains remained mostly in blastoconidia form, consistent with the faster growth rate of resistant strains. Notably, 20% ALA-PDT effectively inhibited biofilm formation in both sensitive and drug-resistant *C. albicans*, highlighting its potential to disrupt key virulence factors associated with antifungal resistance.

### Pretreatment with ALA-PDT increased the susceptibility of drug-resistant strains to previously ineffective antifungal agents

**Fig 2:**
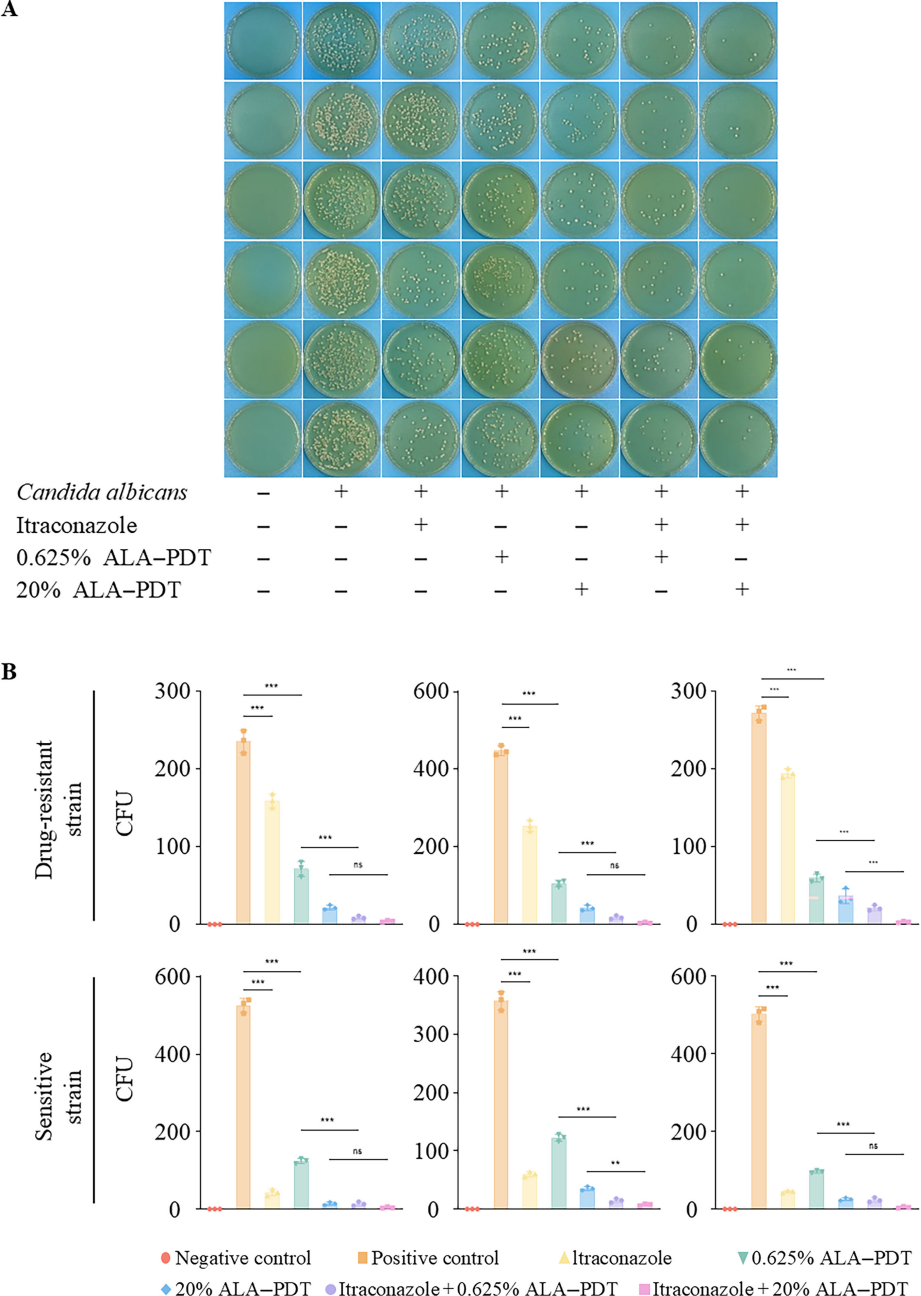
Synergistically equal antifungal effects of 5-aminolevulinic acid photodynamic therapy (ALA-PDT) and itraconazole against susceptible and drug-resistant *C. albicans*. (**A**) Representative agar plates showing fungal growth of drug-sensitive and drug-resistant *C. albicans* under different treatment conditions. (**B**) Quantitative analysis of colony-forming units (CFU) from three independent experiments. Both 0.625% and 20% ALA-PDT treatments reduced fungal burden compared to untreated controls. The combination of ALA-PDT with itraconazole resulted in significantly lower CFU counts than either treatment alone in both resistant and sensitive strains. Notably, the fungal load showed no significant difference between resistant and sensitive strains under combined ALA-PDT and itraconazole treatment, indicating a restored antifungal susceptibility. *P* > 0.05; * *P* ≤ 0.05; ** *P* ≤ 0.01; *** *P* ≤ 0.001.

The antifungal effects of ALA-PDT were further evaluated in both drug-resistant and drug-sensitive *C. albicans* strains. Pretreatment with ALA-PDT increased the susceptibility of drug-resistant strains to previously ineffective antifungal agents. Given that *Candida* spp. commonly exhibit resistance to terbinafine and, to a lesser extent, itraconazole, we focused on these two drugs to assess combined efficacy with ALA-PDT. The results showed that pre-ALA-PDT combined with terbinafine enhanced inhibition of *C. albicans* growth compared to ALA-PDT alone (Fig. 2). Specifically, both 0.625% and 20% ALA-PDT significantly reduced fungal burden compared to antifungal drugs alone in resistant and sensitive strains, and the combined treatment further decreased fungal survival relative to ALA-PDT alone.

In contrast, combining ALA-PDT with itraconazole did not produce a significant difference in fungal burden between treated resistant and sensitive strains. Overall, these findings demonstrate that ALA-PDT can effectively enhance the antifungal activity of certain drugs, particularly terbinafine, against *C. albicans* strains with intrinsic terbinafine resistance.

### ALA-PDT elevates porphyrins and ROS to promote killing of *C. albicans*

**Fig 3:**
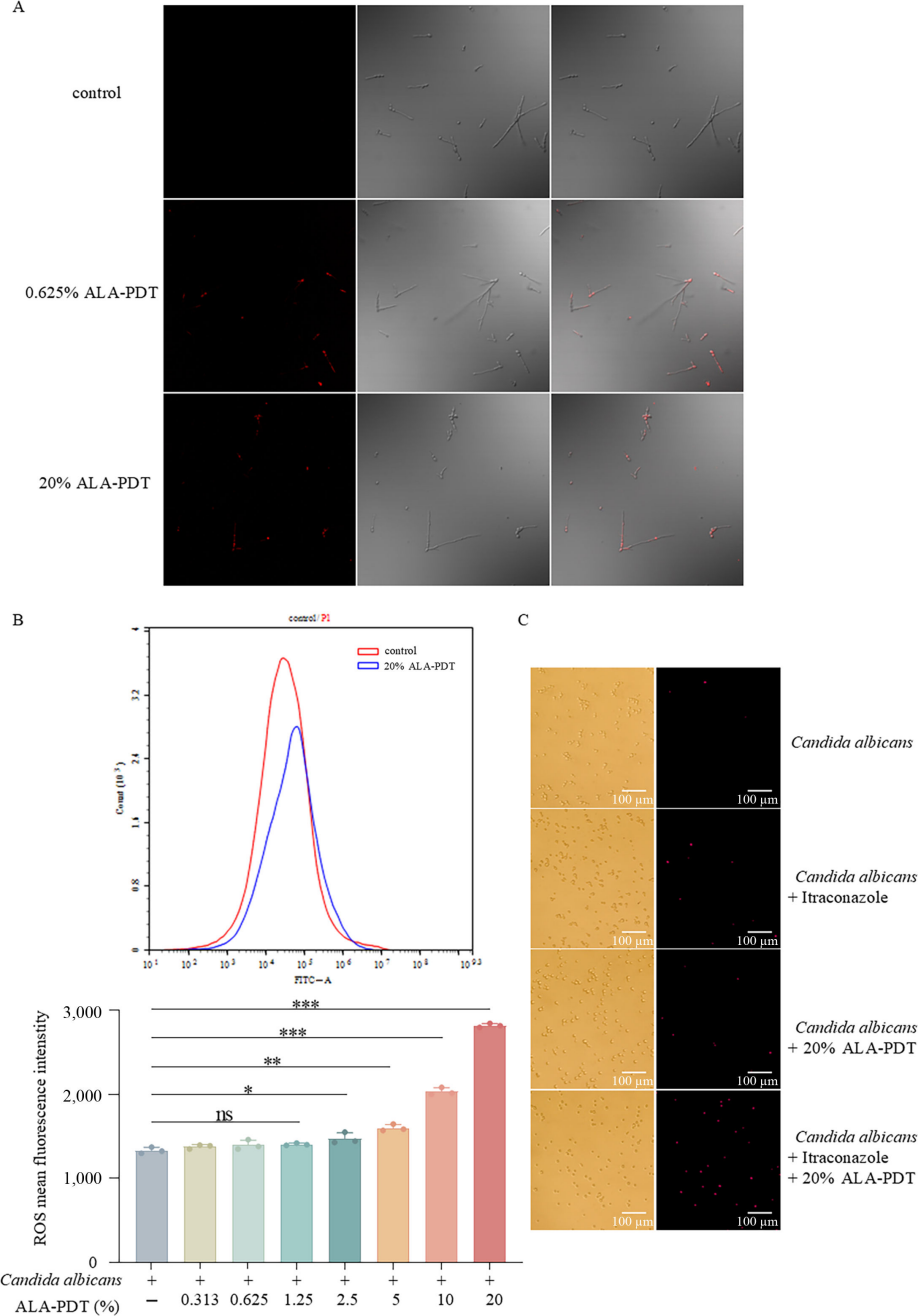
5-Aminolevulinic acid photodynamic therapy (ALA-PDT) promotes reactive oxygen species (ROS) generation and disrupts cell membrane integrity in *C. albicans*.* Candida* strains were treated with ALA alone, PDT alone, or ALA-PDT, with untreated cells serving as the control. (**A**) Confocal microscopy analysis showing porphyrin accumulation. Strong red fluorescence in ALA-PDT–treated groups indicates increased intracellular porphyrin levels compared with groups treated with ALA alone, light irradiation alone, or medium control. (**B**) Quantification of intracellular ROS levels in untreated and ALA-PDT–treated *C. albicans*. ALA-PDT markedly elevated ROS levels relative to all control groups. (**C**) Propidium iodide (PI) staining analysis of membrane integrity. Minimal red fluorescence was observed in the control group, whereas both itraconazole and ALA-PDT treatments slightly increased PI fluorescence. The combined ALA-PDT and itraconazole treatment produced the strongest red fluorescence, indicating severe membrane damage and loss of cell integrity. *P* > 0.05; * *P* ≤ 0.05; ** *P* ≤ 0.01; *** *P* ≤ 0.001.

We used laser confocal microscopy to examine porphyrin accumulation within mitochondria. *C. albicans* treated with ALA-PDT exhibited markedly stronger red fluorescence, indicative of porphyrin accumulation, compared to untreated controls (Fig. 3A), demonstrating that ALA-PDT effectively enhances intracellular porphyrin production.

Consistent with the increased porphyrin levels and more efficient fungal killing, ALA-PDT–treated *C. albicans* also showed a significant increase in intracellular ROS compared to untreated strains (Fig. 3B). ROS levels did not differ significantly among groups treated with culture medium alone, ALA alone, or red-light irradiation alone. These results indicate that ROS production induced by ALA-PDT is a key contributor to the killing of *C. albicans*.

### ALA-PDT combined with itraconazole affects the membrane integrity of *C. albicans*

Azole drugs target the fungal cell membrane. In this experiment, PI staining was used to assess membrane integrity of *C. albicans* following treatment with itraconazole alone or in combination with ALA-PDT. As shown in Fig. 3C, the control group exhibited no red fluorescence. There was no significant difference in red fluorescence intensity between the itraconazole-only and ALA-PDT-only groups, although both showed slightly increased fluorescence compared to the control. Notably, the combination of ALA-PDT and itraconazole produced markedly higher red fluorescence than all other groups, indicating enhanced membrane permeability, which may contribute to fungal cell apoptosis and the observed abnormal cell morphology.

### ALA-PDT treatment significantly alters mycelia-related gene expression

**Fig 4:**
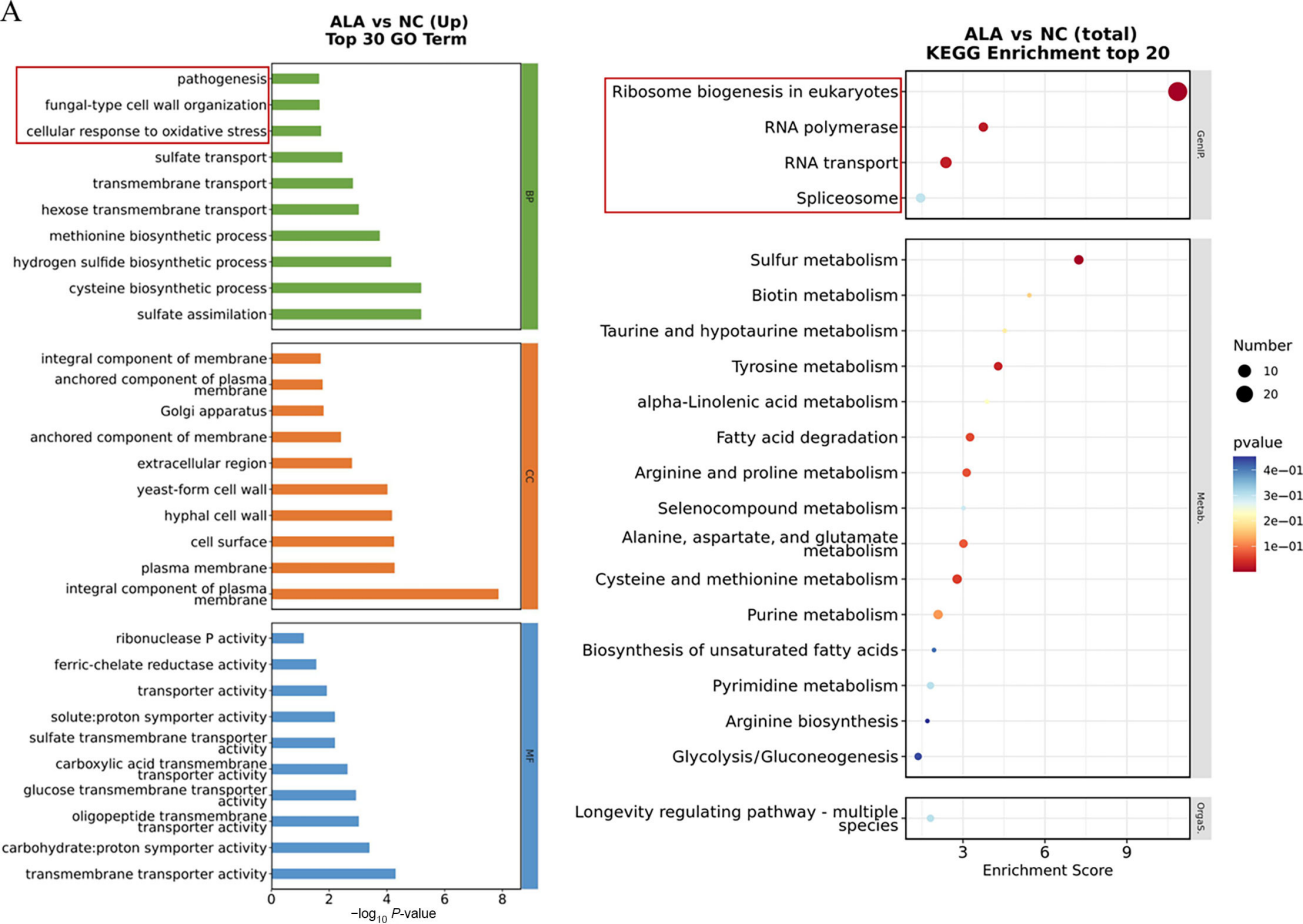
Transcriptomic and gene expression analyses of 5-aminolevulinic acid photodynamic therapy (ALA-PDT)–treated *Candida albican*s. Kyoto Encyclopedia of Genes and Genomes and Gene Ontology enrichment analyses revealed that ALA-PDT pretreatment significantly altered multiple RNA transcription–related pathways compared with untreated *C. albicans*. (**A**) The GO Term analysis revealed that after ALA-PDT treatment, the differentially expressed genes were significantly enriched in cell wall structure remodeling, oxidative stress defense, and modification of cell membrane components, directly correlating with the regulation of bacterial pathogenicity. The KEGG enrichment analysis showed that after ALA-PDT treatment, the differentially expressed genes were highly enriched in ribosome/RNA metabolism and fatty acid metabolism pathways.

**Fig 5:**
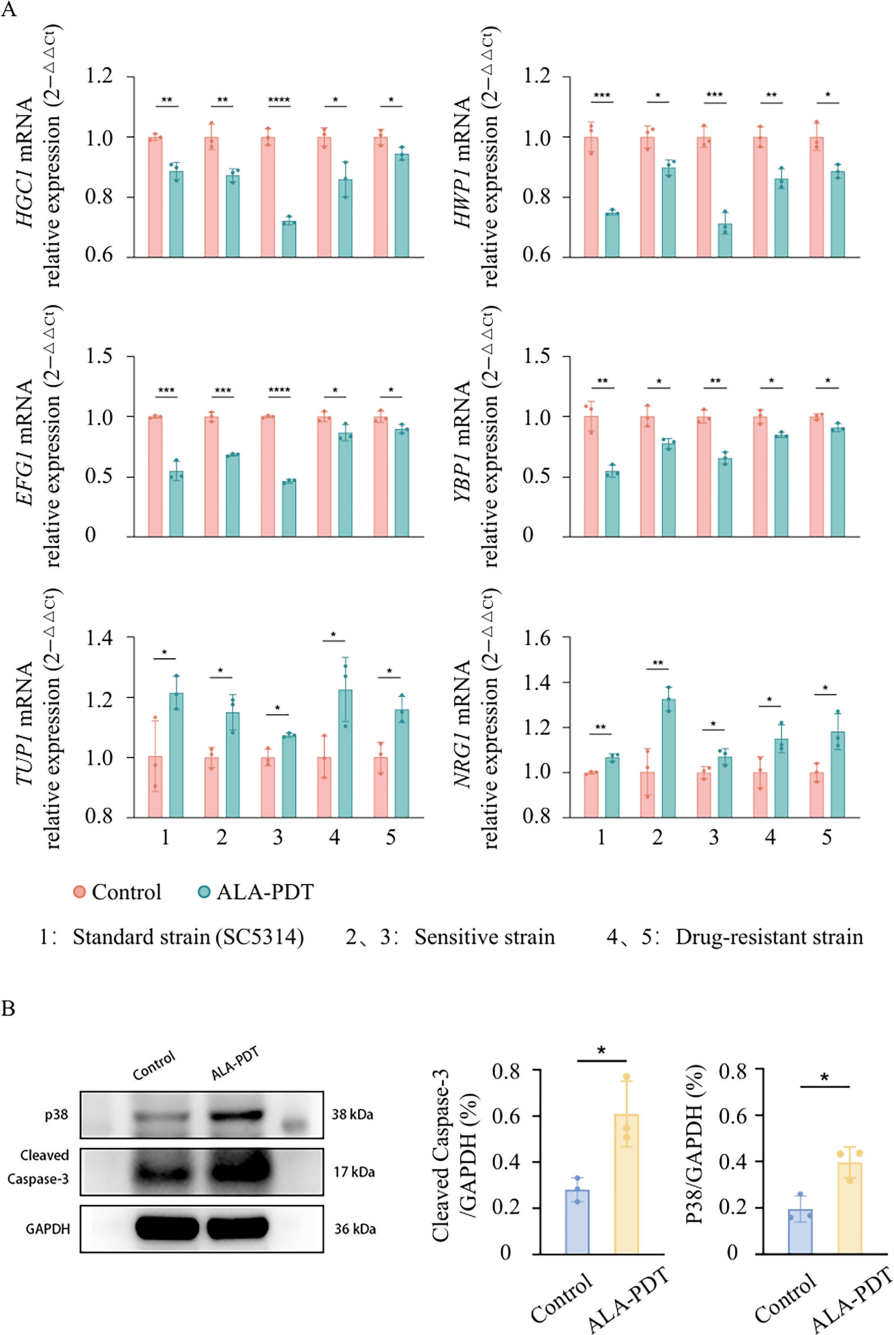
RT-qPCR and western blot (WB) validation of mycelia-associated gene expression. The standard strain SC5314, azole-susceptible strains 517 (*Δece1*) and CHK21 (*chk1Δ*), (marked sensitive 2, 3) and their corresponding reconstituted strains 55 and CHK23 (marked as drug-resistant strain) were analyzed. The expression levels of *HGC1*, *HWP1*, *EFG1*, and *YBP1* were markedly downregulated in the 5-aminolevulinic acid photodynamic therapy (ALA-PDT)–treated group compared with untreated controls, in both mycelial-positive and mycelial-defective strains. The WB results showed that the P38 and cleaved caspase-3 in the ALA-PDT treatment group were higher than those in the control group. (**A**) After ALA-PDT treatment, the mRNA expression levels of genes related to hyphal formation and pathogenicity, such as HGC1, HWP1, EFG1, and YBP1, in the standard strain, drug-sensitive strain, and drug-resistant strain of SC5314 were all significantly downregulated. This suggests that ALA-PDT can inhibit the occurrence of fungal hyphae and weaken their pathogenic ability by regulating the expression of these genes. (**B**) After ALA-PDT treatment, the relative expression levels of Cleaved Caspase-3 and stress-related protein P38 in the standard strain, drug-sensitive strain, and drug-resistant strain of SC5314 were all significantly increased, indicating that ALA-PDT can regulate the activation of the cell apoptosis pathway and stress pathway. *P* > 0.05; * *P* ≤ 0.05; ** *P* ≤ 0.01; *** *P* ≤ 0.001

Transcriptome analysis revealed notable differences in gene expression between 0.625% ALA-PDT–treated *C. albicans* and untreated controls (Fig. 4). Kyoto Encyclopedia of Genes and Genomes (KEGG) and gene ontology (GO) pathway analyses indicated significant alterations in RNA transcription pathways following ALA-PDT pretreatment. To further explore the mechanisms underlying ALA-PDT’s effects, we validated genes associated with hyphal growth by RT-PCR method. In consistency with RNA-seq results, RT-PCR analysis revealed that expression levels of *HGC1*, *HWP1*, *EFG1*, and *YBP1* were significantly downregulated in the ALA-PDT–treated group compared to untreated *C. albicans* (Fig. 5A), suggesting that ALA-PDT inhibits hyphal development.

Additionally, to confirm the apoptotic effect following ALA-PDT treatment, we assessed proteins involved in stress response and apoptosis. The levels of p38, a key mediator of cellular stress responses, and cleaved caspase-3, a core apoptosis marker, were significantly elevated in the ALA-PDT–treated group compared to controls (Fig. 5B). These findings indicate that ALA-PDT not only suppresses hyphal growth but also promotes stress-induced apoptosis in *C. albicans*.

## DISCUSSION

Antifungal drugs remain the mainstay of candidiasis treatment, but repeated and prolonged use increases the risk of drug resistance (22, 23) and drug-related toxicity, particularly since azoles are primarily fungistatic (24). The emergence of drug-resistant *Candida* spp. strains in vulnerable populations, such as the patient cohort in this study, exacerbates the challenges of managing candidiasis (25). Consistent with previous reports, we found that 23.79% (49/206) of *Candida* spp. isolates were drug-resistant, with a notable prevalence among elderly patients in the ICU. These patients often present with underlying conditions, including compromised immunity, prolonged hospitalization, and intensive treatments, which predispose them to repeated infection events. These findings highlight the urgent need for alternative therapeutic strategies, such as ALA-PDT, for managing drug-resistant *Candida* spp. infections in elderly ICU patients.

*C. albicans* is the most virulent and common species within the *Candida* spp. genus. In healthy individuals, it forms a benign biofilm on mucosal surfaces (26), but it can transition to a pathogenic state under conditions such as mucosal barrier disruption, immune imbalance, or prolonged antibiotic therapy (24, 27). Several clinical features characterize the isolates in our study cohort. First, drug-resistant *Candida tropicalis* and *C. albicans* accounted for over two-thirds of resistant strains. Second, the majority of patients were elderly (≥60 years: 73.47%) in the ICU. Among them, 58.7% (121/206) had two or more chronic diseases, mainly involving the respiratory system (e.g., chronic obstructive pulmonary disease and pulmonary interstitial fibrosis), circulatory system (e.g., Grade 3 hypertension and coronary atherosclerotic heart disease), and metabolic system (e.g., type 2 diabetes and chronic renal failure), creating favorable conditions for *Candida* spp. colonization and the development of drug resistance. Third, 12.1% (25/206) of patients were receiving immunosuppressive therapy, including glucocorticoids (e.g., prednisone) for rheumatic immune diseases such as rheumatoid arthritis or dermatomyositis. These drugs can directly impair key immune clearance pathways against *C. albicans* by inhibiting T-cell activation and reducing antibody production (28).

Our findings also indicate that drug-resistant strains exhibit faster growth and blastoconidia germination than drug-sensitive strains. Clinically, this underscores the importance of early intervention: antifungal therapy should ideally be administered before blastoconidia germination to prevent hyphal formation and biofilm development, thereby improving therapeutic outcomes.

ALA-PDT has been explored for treating a variety of diseases, including infections caused by bacteria, viruses, fungi, and other microorganisms (29–31). Its antifungal potential has been demonstrated in multiple clinical studies, such as those on persistent chromoblastomycosis, where combining ALA-PDT with antifungal agents offered a compelling alternative to conventional therapies (32, 33). In addition, previous reports suggest that ALA-PDT can enhance the effectiveness of antifungal agents against *C. albicans (*34). Collectively, these studies confirm that both ALA-PDT and antifungal agents can inhibit *C. albicans*, with their combination often yielding synergistic effects. Importantly, in this study, we observed that ALA-PDT pretreatment increased the susceptibility of *C. albicans*, including drug-resistant strains, to antifungal drugs, suggesting that sequential application of ALA-PDT followed by antifungal therapy may provide a novel strategy for treating *Candida* spp. infections, particularly those caused by drug-resistant strains.

**Fig 6:**
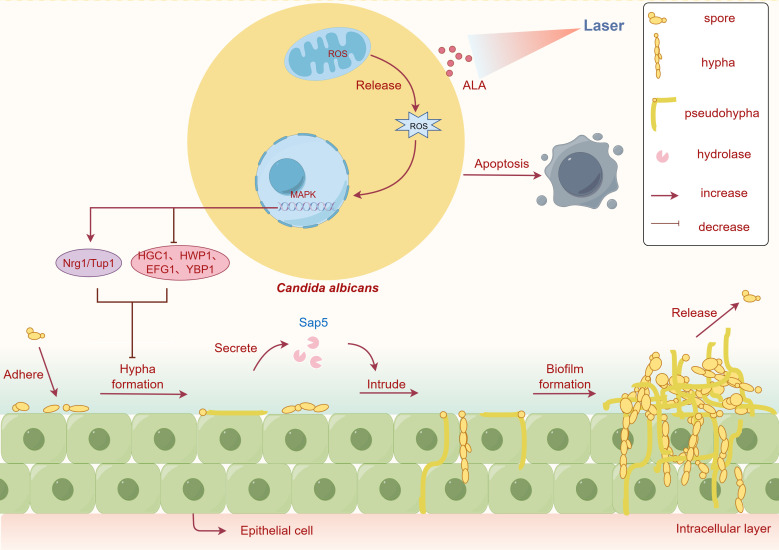
Proposed mechanism of 5-aminolevulinic acid photodynamic therapy (ALA-PDT)–mediated antifungal activity against *Candida albicans*. ALA-PDT induces the generation of reactive oxygen species (ROS), leading to oxidative stress that disrupts mitochondrial function and cell membrane integrity. Elevated ROS levels downregulate the expression of mycelial growth–related genes (*HGC1, HWP1, EFG1,* and *YBP1*) while upregulating negative regulatory genes, thereby inhibiting hyphal development and biofilm formation. These combined effects result in membrane cracking, apoptosis, and effective elimination of *C. albicans* cells.

Despite these promising results, the application of ALA-PDT against drug-resistant *Candida* spp. *r*emains limited, and the underlying mechanisms have not been fully elucidated. In our study, 20% ALA-PDT effectively killed *C. albicans*, and its combination with antifungal agents enhanced antifungal sensitivity, disrupted biofilms, and significantly reduced fungal burden. Mechanistically, ALA-PDT induced ROS generation, activated the P38 stress response, and triggered apoptosis-associated cell membrane damage. ROS generated by ALA-PDT further activated the MAPK pathway in *C. albicans*, impairing mitochondrial function and compromising cell membrane integrity. This oxidative stress may directly influence the expression of mycelia-related genes: *HGC1*, *HWP1*, *EFG1*, and *YBP1*, which promote hyphal formation, were downregulated, whereas the negative regulatory genes *NRG1* and *TUP1* were upregulated. These transcriptional changes then inhibited the transition of blastoconidia to hyphae or pseudohyphae, thereby suppressing biofilm formation. Likely, elevated ROS caused irreversible damage to multiple microbial targets, including membrane disruption, protein oxidation, and nucleic acid damage, ultimately leading to *C. albicans* apoptosis and effective fungal clearance (Fig. 6).

The antibody we used in this study is a widely validated cross-reactive antibody that specifically recognizes the conserved phosphorylated activation motif of p38. This antibody has been successfully applied in published studies focusing on *C. albicans* stress signaling (35).

In summary, our study highlights the efficacy of ALA-PDT in directly targeting and killing drug-resistant *Candida* spp. This therapeutic approach induces the formation of reactive oxygen species (ROS), which inhibit hyphal growth and promote fungal cell death through non-specific oxidative mechanisms. The rapid growth rate of drug-resistant *C. albicans* strains may contribute to their resistance, suggesting that interventions aimed at regulating growth can enhance treatment efficacy. ALA-PDT effectively overcomes these resistance mechanisms and has the potential to improve clinical outcomes in patients with drug-resistant *Candida* spp. infections. Whether used alone or in combination with antifungal agents, ALA-PDT represents a promising alternative strategy for the treatment of *Candida* spp. infections, particularly those caused by drug-resistant strains.

## Data Availability

The data presented in the study are included in the article and its supplemental material; further inquiries can be directed to the corresponding authors.
